# Quercetin inhibits virulence properties of *Porphyromas gingivalis* in periodontal disease

**DOI:** 10.1038/s41598-020-74977-y

**Published:** 2020-10-27

**Authors:** Zhiyan He, Xu Zhang, Zhongchen Song, Lu Li, Haishuang Chang, Shiliang Li, Wei Zhou

**Affiliations:** 1 Laboratory of Oral Microbiota and Systemic Diseases, Shanghai Ninth People’s Hospital, College of Stomatology, Shanghai Jiao Tong University School of Medicine, Shanghai, China; 2 National Clinical Research Center for Oral Diseases, Shanghai, China; 3 Shanghai Key Laboratory of Stomatology & Shanghai Research Institute of Stomatology, Shanghai, China; 4 Department of Periodontology, Shanghai Ninth People’s Hospital, College of Stomatology, Shanghai Jiao Tong University School of Medicine, Shanghai, China; 5 Shanghai Key Laboratory of New Drug Design, State Key Laboratory of Bioreactor Engineering, School of Pharmacy, East China University of Science and Technology, Shanghai, China; 6 Shanghai Institute of Precision Medicine, Shanghai Ninth People’s Hospital, Shanghai Jiao Tong University School of Medicine, Shanghai, China

**Keywords:** Chemical biology, Microbiology

## Abstract

*Porphyromonas gingivalis* is a causative agent in the onset and progression of periodontal disease. This study aims to investigate the effects of quercetin, a natural plant product, on *P. gingivalis* virulence properties including gingipain, haemagglutinin and biofilm formation. Antimicrobial effects and morphological changes of quercetin on *P. gingivalis* were detected. The effects of quercetin on gingipains activities and hemolytic, hemagglutination activities were evaluated using chromogenic peptides and sheep erythrocytes. The biofilm biomass and metabolism with different concentrations of quercetin were assessed by the crystal violet and MTT assay. The structures and thickness of the biofilms were observed by confocal laser scanning microscopy. Bacterial cell surface properties including cell surface hydrophobicity and aggregation were also evaluated. The mRNA expression of virulence and iron/heme utilization was assessed using real time-PCR. Quercetin exhibited antimicrobial effects and damaged the cell structure. Quercetin can inhibit gingipains, hemolytic, hemagglutination activities and biofilm formation at sub-MIC concentrations. Molecular docking analysis further indicated that quercetin can interact with gingipains. The biofilm became sparser and thinner after quercetin treatment. Quercetin also modulate cell surface hydrophobicity and aggregation. Expression of the genes tested was down-regulated in the presence of quercetin. In conclusion, our study demonstrated that quercetin inhibited various virulence factors of *P. gingivalis*.

## Introduction

Periodontal disease is a common chronic inflammatory disease that characterized swelling and bleeding of the gums clinically, and leading to the progressive destruction of tooth-supporting tissues including the gingiva, alveolar bone, periodontal ligament, and cementum. Severe periodontitis can cause periodontal pockets formation, bone resorption, and eventually lead to tooth loss^1^. It has been estimated that nearly 50% of human population worldwide are affected by mild to moderate periodontitis and approximately 11% are severe forms^2^. In addition to the oral local sequelae, evidence has accumulated to suggest that periodontal disease is also related to a risk factor for systemic complications such as cardiovascular diseases, stroke, diabetes, arthritis, and preterm low birth weight for more than a decade^3,4^.

Two major etiological factors of periodontal disease are specific bacterial species called periodontopathogens colonizing subgingival sites, and subsequent host inflammatory and immune responses to these periodontopathogens^5^. Although over 700 microbial species have been identified in subgingival plaque, *Porphyromonas gingivalis*, a black-pigmented, rod-shaped, and Gram-negative anaerobic bacterium, is a keystone pathogen for periodontal disease^6^.

*P. gingivalis* possesses various potential virulence factors including gingipain proteases, haemagglutinin, fimbriae, capsule, lipopolysacharides and major outer-membrane proteins to evade the host immune defense system and destroy host connective tissues^7,8^. One major virulence factor is gingipain proteases consist of lysine-specific protease (Lys-gingipain (Kgp)) and arginine-specific protease (Arg-gingipain (Rgp)). Kgp is encoded by *kgp* gene and Rgp is further subdivided into RgpA and RgpB, encoded by *rgpA* and* rgpB* genes, respectively^9,10^. Hemagglutinin allowed *P. gingivalis* to adhere to host cells, which is an initial step in bacterial infection process. It facilitates the acquisition of heme through erythrocyte binding, causes lysis and aggregation of erythrocytes^11^. Besides, *P. gingivalis* is a late colonizer of subgingival plaque biofilm, and adheres to plaque-colonizing organisms including *Streptococcus gordonii* and *Fusobacterium nucleatum*^12^. A biofilm is a community of bacteria that attached to the surface and in a self-produced extracellular polymeric substances matrix consisting of protein, polysaccharides and nucleic acid^13^.

Therefore, the inhibition of *P. gingivalis* virulent effects may impede progression of periodontitis, prevent and control periodontal diseases. In the last decade, natural polyphenols have been studied since they have various biological functions such as antimicrobial, anti-inflammation, anti-oxidation, and anti-cancer. The largest class of polyphenols is flavonoids which have two aromatic rings linked with through three carbon atoms that form an oxygenated heterocycle. Quercetin (3,3′,4′,5,7-pentahydroxyflavone) is the most abundant flavonoid that exists in various vegetables and fruits including apples, tea, onions, red wine, red grapes, berries and tomatoes^14,15^. Quercetin has been reported to be effective against Gram-positive and Gram-negative bacteria, such as *Staphylococcus aureus*, *Escherichia coli* and *Pseudomonas aeruginosa*^16–18^. However, to our knowledge there are few studies focused on the influence of quercetin on *P. gingivalis* virulence factors. Therefore, in the present study, our aim was to determine the effect of quercetin on *P. gingivalis* virulent pathogenicity with respect to periodontal disease.

## Results

### Antimicrobial activity of quercetin against *P. gingivalis* planktonic

**Figure 1:**
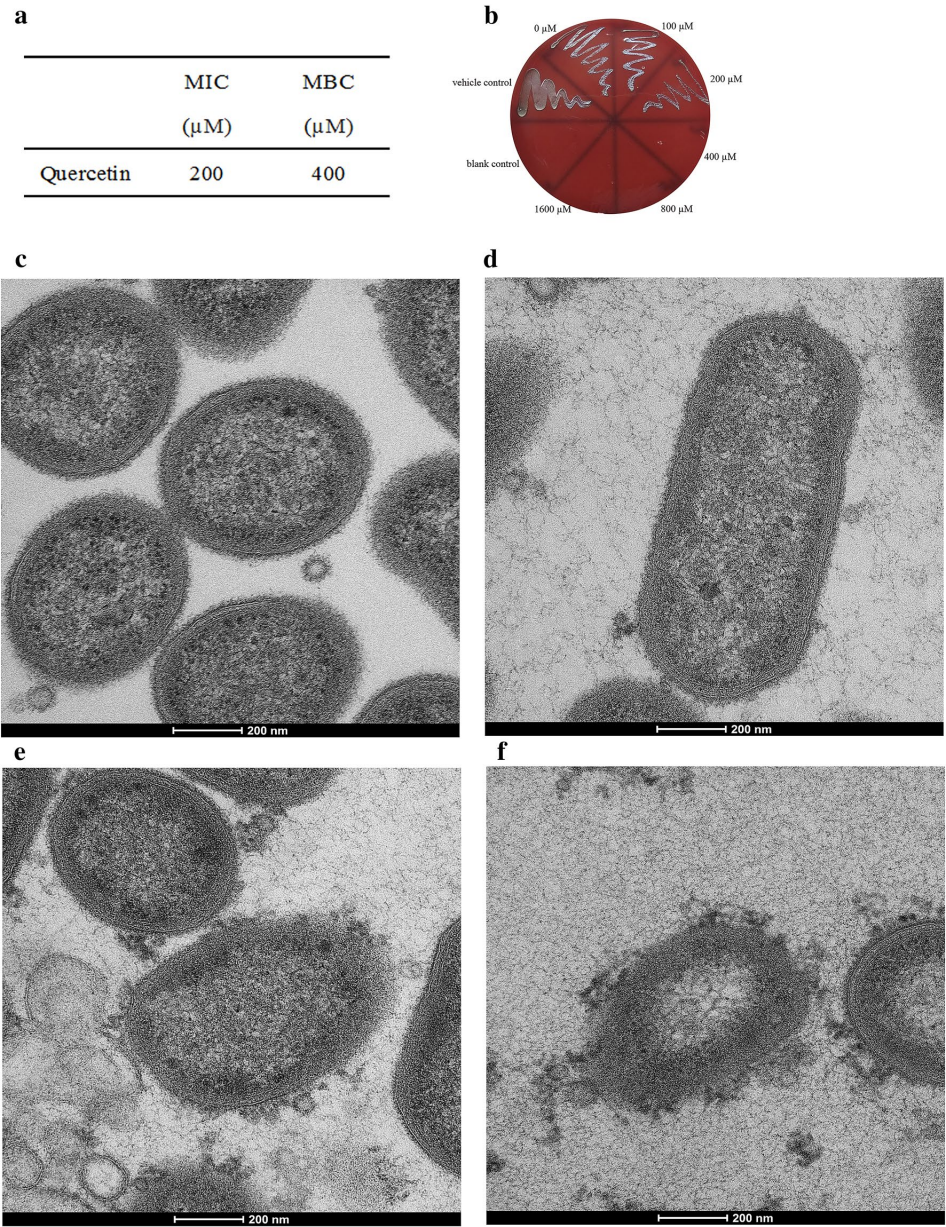
Antibacterial activity and morphological changes of *P. gingivalis*. (**a**) MIC and MBC values of quercetin against planktonic *P. gingivalis*. 10^7^ CFU/mL *P. gingivalis* suspension was added in a flat-bottomed 96-well microplate at final concentrations of 0–1600 μM quercetin and incubated under anaerobic conditions at 37 °C. (**b**) The number of colonies on blood agar. An aliquot of 10 μL cell suspension from each well was taken from above 96-well microplate, and bacterial clones were counted on the blood agar plates after incubation for 3–5 days. (**c**–**f**) TEM images of *P. gingivalis* treated with different concentration of quercetin. *P. gingivalis* cells with different concentrations of quercetin cultured at 37 °C for 4 h. The cell pellets were fixed with 2.5% glutaraldehyde at 4 °C, exposed to 2% osmium tetraoxide for 2 h, dehydrated in a series of ethanol and dried in acetone. Then the samples were embedded in resin blocks, cut into ultrathin sections, and stained with uranyl acetate and lead citrate. (**c**) 0 μM, (**d**) 100 μM, (**e**) 200 μM, (**f**) 400 μM, Bar = 200 nm.

The antibacterial activity of quercetin against *P. gingivalis* was determined with a broth microdilution assay. Results of the susceptibility assay of *P. gingivalis* planktonic cultures to quercetin are shown in Fig. 1a,b. The MIC and the MBC values of quercetin were 200 and 400 μM, respectively.

### Observation of morphological changes

The morphological changes of *P. gingivalis* treated with different concentrations of quercetin was observed by TEM. The cell membranes remained clearly intact in the control group (Fig. 1c). The structures of bacteria cells differ with final concentrations of 100, 200, and 400 μM quercetin (Fig. 1d–f). We saw considerably damage and discontinuity of cell membrane and the cell structures were damaged with increasing severity as concentration of quercetin increased. Thus, quercetin caused integrity loss of the cell membrane.

### Effect of quercetin on gingipain activities

**Figure 2:**
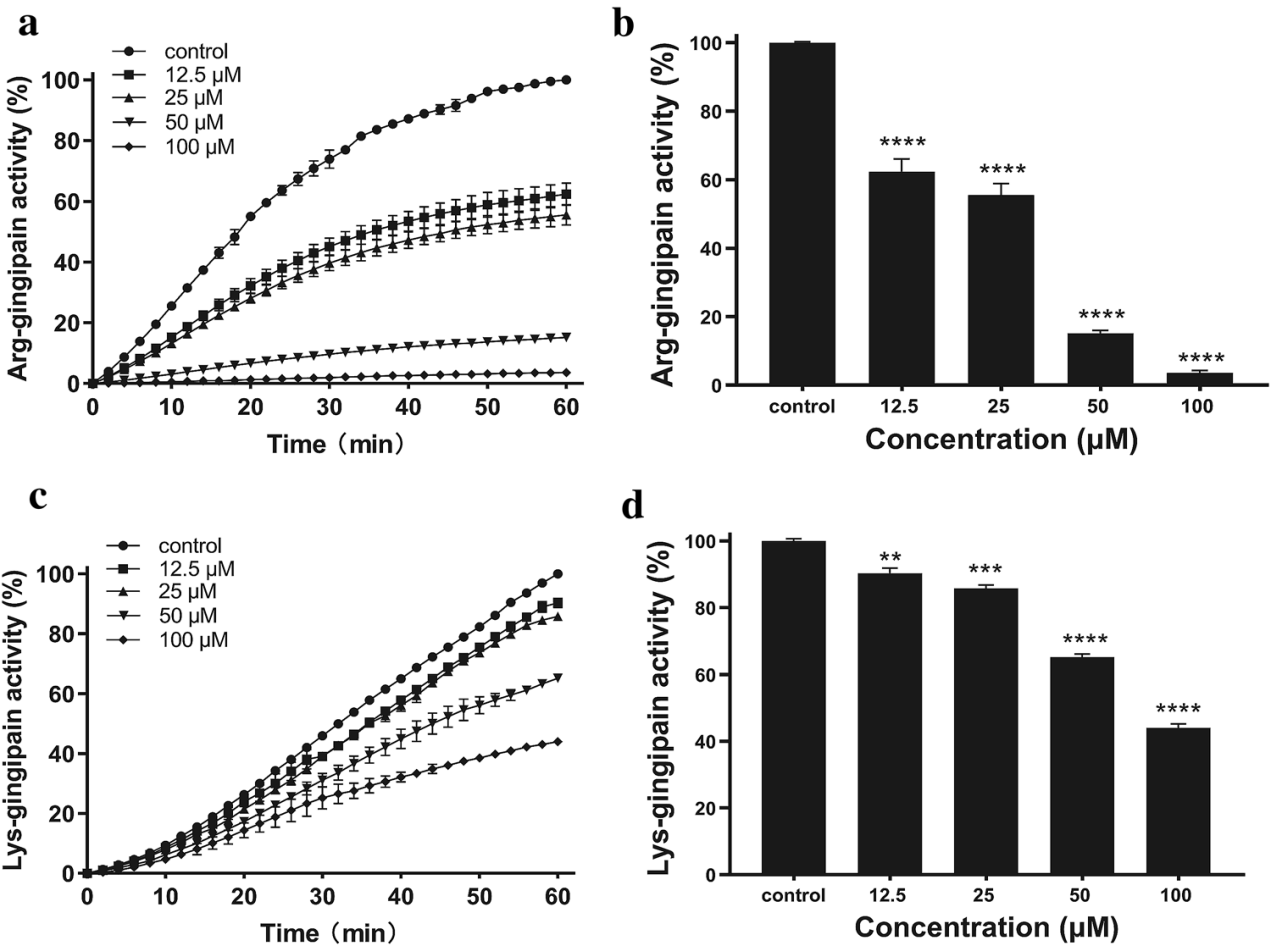
Effect of quercetin on Arg-gingipain (Rgp) and Lys-gingipain (Kgp) activity. *P. gingivalis* culture was harvested and suspended in PBS to OD_660nm_ of 2 for Rgp activity and of 1 for Kgp activity. The *P. gingivalis* cells were incubated in PBS with or without quercetin at 37 °C in the dark for 3 h. Activity of Rgp (**a**,**b**) and Kgp (**c**,**d**) indicated as the hydrolysis of the specific chromogenic substrates (BAPNA and ALNA) was detected at every 2 min for 1 h by measuring the absorbance at 405 nm. The inhibition of substrate degradation was also measured as a function of time (**b**,**d**). Bars denoted by (****) indicate significant difference at *p* < 0.0001 by one-way analysis of variance (ANOVA) with Dunnett’s post hoc test.

The activities of Rgp and Kgp influenced by quercetin at sub-MIC concentrations were measured. Quercetin inhibited Rgp and Kgp activities in dose-dependent manners significantly. The inhibitory effects within 1 h are also shown in Fig. 2a,c. The inhibition rates on Kgp activities decreased from 90.33% at 12.5 μM to 43.97% at 100 μM, as shown in Fig. 2b. The inhibition rates on Rgp activities decreased from 62.36% at 12.5 μM to 3.64% at 100 μM, as shown in Fig. 2d.

### Binding mode analysis

**Figure 3:**
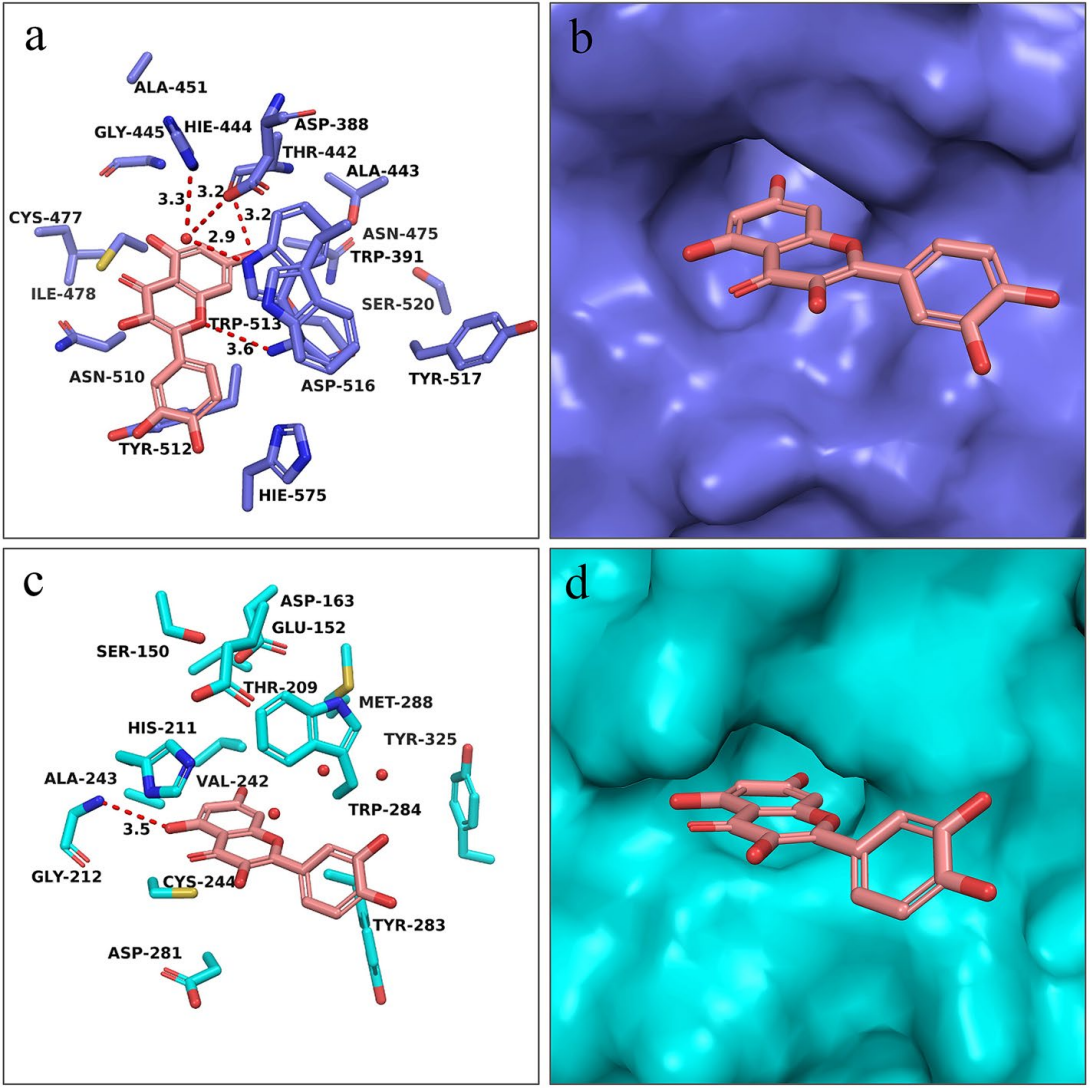
Predicted binding modes of Quercetin in the binding pocket of Kgp (**a**,**b**, PDB ID: 6I9A) and RgpB (**c**,**d**, PDB ID: 1CVR). Quercetin is shown as pink sticks. Key residues around the binding pocket are displayed as sticks in (**a**) (Kgp, purple) and (**c**) (RgpB, blue). Hydrogen bonds are highlighted as red dashed lines, and water is depicted as small red balls. The surfaces of the substrate binding site in Kgp and RgpB are shown in (**b**) (purple) and (**d**) (blue), respectively.

The substrate binding site of Kgp was reported to contain a catalytic triad including residues Cys477, His444 and Asp388^19^, which may form a charge-relay system for catalysis. Compared with Kgp, the catalytic triad of RgpB is Cys244, His211 and Glu152^20^. Quercetin was docked into active sites of Kgp and RgpB by using Glide^21^. The results showed that Quercetin could bind to the substrate binding site of Kgp and RgpB with favorable intermolecular interactions. In the predicted binding mode of quercetin to Kgp (Fig. 3a), the almost planar quercetin core accommodates the substrate binding site well by shape matching and forming two hydrogen bonds with residues Ala443O and Trp513N. Similarly, in the predicted binding mode of quercetin to RgpB (Fig. 3c), quercetin core also occupies the substrate binding site well and could form a hydrogen bond with residue Gly212N. When showing the surface of Kgp (Fig. 3b) and RgpB (Fig. 3d), it is observed that quercetin core could penetrate into the usually -Lys- and -Arg- fragments binding cavity of Kgp and RgpB respectively, which is beneficial for preventing the substrate from getting close and thus inhibiting the catalytic activity of the two enzymes.

### Effect of quercetin on hemagglutination and hemolytic activity

**Figure 4:**
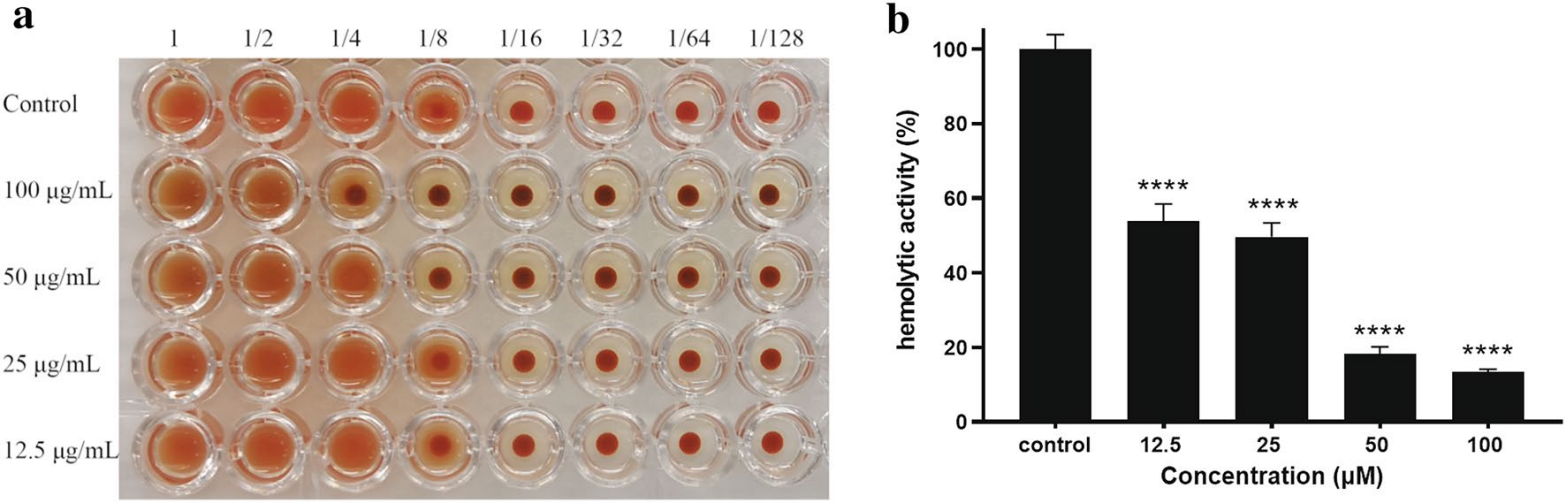
Effect of quercetin on hemagglutination and hemolytic activity. (**a**) Overnight cultures of *P. gingivalis* with quercetin were harvested, and suspended in PBS. 100 μL of PBS was added to each well in a 96-well round-bottom microtiter plate, 100 μL of the bacterial cells was suspended in PBS, added to the first well and serially diluted (1:2 to 1:128). Sheep erythrocytes suspension (100 μL, 2% in PBS) and quercetin were added to each well, and the plate was incubated for 3 h. Hemagglutination activities were evaluated visually. (**b**) *P. gingivalis* cells were centrifuged, re-suspended to a final OD_600nm_ of 1.5. The sheep erythrocytes at a concentration of 1% were mixed with an equal volume of bacterial cells with or without quercetin at 37 °C for 18 h. The hemolytic activity was determined at wavelength of 405 nm. Bars denoted by (****) indicate significant difference at *p* < 0.0001 by one-way analysis of variance (ANOVA) with Dunnett's post hoc test.

Without adding quercetin, hemagglutination was observed up to a dilution of 1:16 for *P. gingivalis*. As shown in Fig. 4a, quercetin showed significantly reduced hemagglutination activity (fourfold for 100 μM and twofold for 50 μM, respectively). But quercetin at lower concentrations (12.5 and 25 μM) had a slight effect on hemagglutination.

Because lysed erythrocytes by bacteria could release hemoglobin, the absorbance of 405 nm for red pigment was used to describe the hemolysis of erythrocytes. The effect of quercetin on the hemolysis of sheep erythrocytes by *P. gingivalis* was determined (Fig. 4b). The hemolytic activities of quercetin (12.5, 25, 50 and 100 μM) were 53.73 ± 4.67%, 49.54 ± 3.76%, 18.23 ± 1.94% and 13.45 ± 0.68%, respectively, compared to the control group. The results indicated that quercetin at sub-MIC concentrations had reduced capacities to lyse erythrocytes.

### Effect of quercetin on biofilm formation

**Figure 5:**
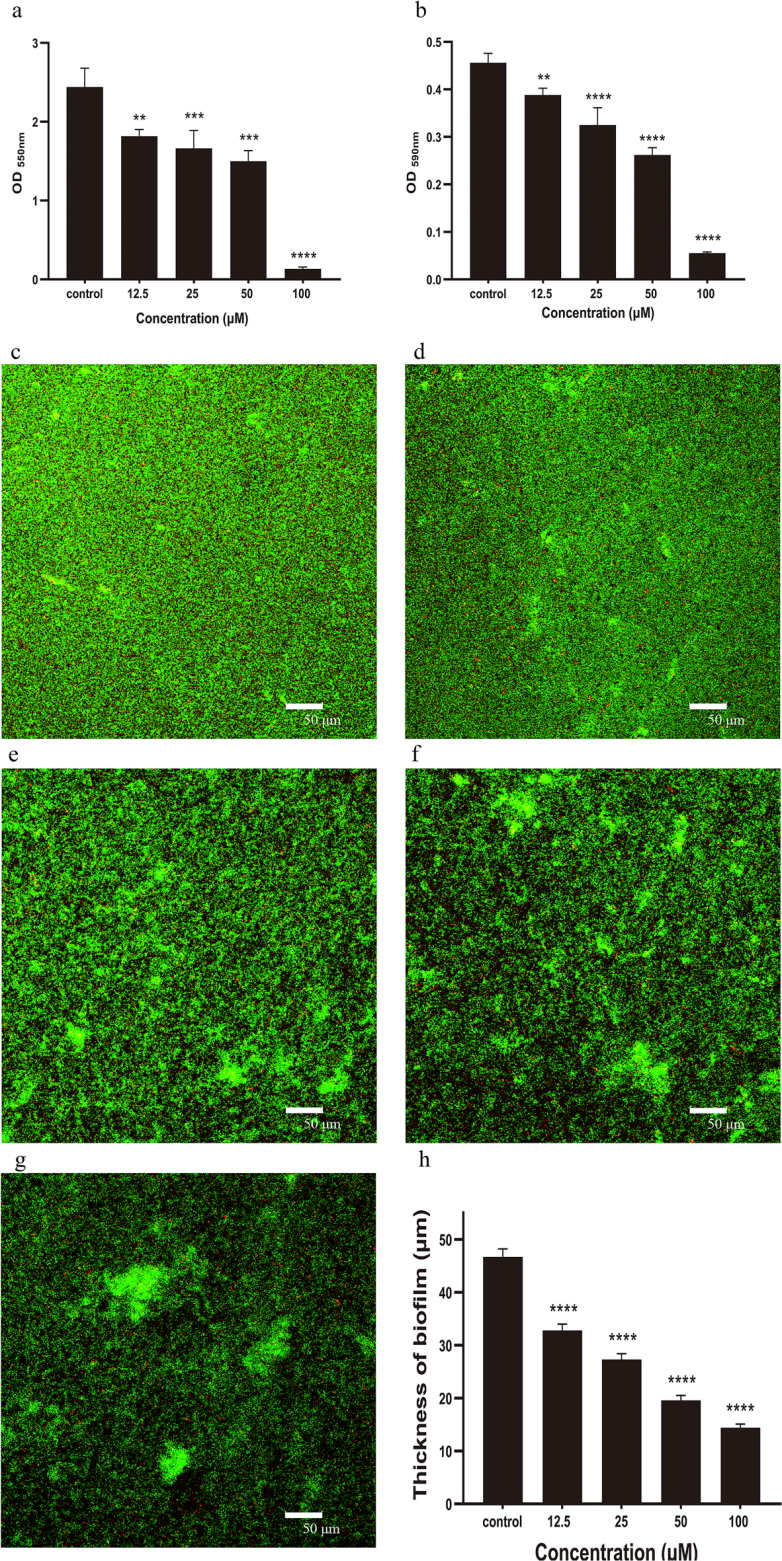
Effect of quercetin on biofilm formation. (**a**) *P. gingivalis* suspension was grown in BHI supplemented with quercetin for 48 h at 37 °C in a 96-well polystyrene plates. After washing three times with sterile PBS, the adherent biofilms were incubated with methanol for 15 min followed by staining with 0.04% (w/v) crystal violet to determine overall biomass of the biofilm at wavelength of 550 nm. (**b**) The biofilm was formed as described for the above method. MTT (0.5 mg/mL) was added to detect the metabolic activity of the biofilms at wavelength of 590 nm. (**c**–**g**) CLSM images of *P. gingivalis* biofilm treated with quercetin. *P. gingivalis* biofilms were formed as described above assay for 48 h on glass coverslips with different concentrations of quercetin. Bacterial cells were washed three times with saline to remove unbound cells and stained with the LIVE/DEAD BacLigh Bacterial Viability Kit containing SYTO 9 dye and propidium iodide. (**c**) 0 μM, (**d**) 12.5 μM, (**e**) 25 μM, (**f**) 50 μM, (**g**) 100 μM, Bar = 50 μm. (**h**) Biofilm thickness. Bars denoted by (**), (***) and (****) indicate significant difference at *p* < 0.01, *p* < 0.001 and *p* < 0.0001, respectively by one-way analysis of variance (ANOVA) with Dunnett's post hoc test.

In addition to growth inhibition of *P. gingivalis* planktonic culture, quercetin also noticeably showed antimicrobial activity against *P. gingivalis* biofilms. The overall biomass of biofilms was quantified by the crystal violet assay with sub-MIC value of quercetin. As shown in Fig. 5a*. P. gingivalis* exhibited OD_550nm_ values of 2.438 ± 0.241, whereas in the presence of quercetin, the values decreased from 1.817 ± 0.083 at 12.5 μM to 0.134 ± 0.022 at 100 μM.

The metabolic activity of biofilms was quantified by the MTT assay with same concentrations of quercetin as above. The metabolic activity of biofilms exhibited OD_590nm_ values with different concentrations of quercetin (12.5, 25, 50, and 100 μM) were 0.388 ± 0.015, 0.325 ± 0.036, 0.262 ± 0.015, and 0.055 ± 0.002, respectively, whereas in the absence of quercetin, the value was 0.456 ± 0.020. The results showed that quercetin decreased the biofilm metabolism compared to the control group and confirmed the crystal violet assay data (Fig. 5b).

### Confocal laser scanning microscopy

To further determine the effect of quercetin on biofilm formation, the structures of the biofilms were observed by CLSM. Live bacteria showed fluorescent green, whereas dead bacteria were fluorescent red. In the absence of quercetin, the biofilm had a highly uniform distribution and dense layer structure (Fig. 5c). As quercetin concentration increased, the biofilm structures were highly dispersed and clearly sparse, and *P. gingivalis* cells tended to aggregate into distinct clusters easily discernable (Fig. 5d–g). In addition, the average thicknesses of biofilms were determined by CLSM (Fig. 5h). The thicknesses of quercetin-treated biofilms were 32.5 ± 0.1 μm at 12.5 μM, 25.6 ± 3.3 μm at 25 μM, 19.8 ± 1.3 μm at 50 μM and 13.3 ± 1.0 μm at 100 μM, respectively, which were thinner than the control group (44.2 ± 2.5 μm). These data confirmed that quercetin inhibited biofilm formation.

### Effect of quercetin on hydrophobicity and aggregation

**Figure 6:**
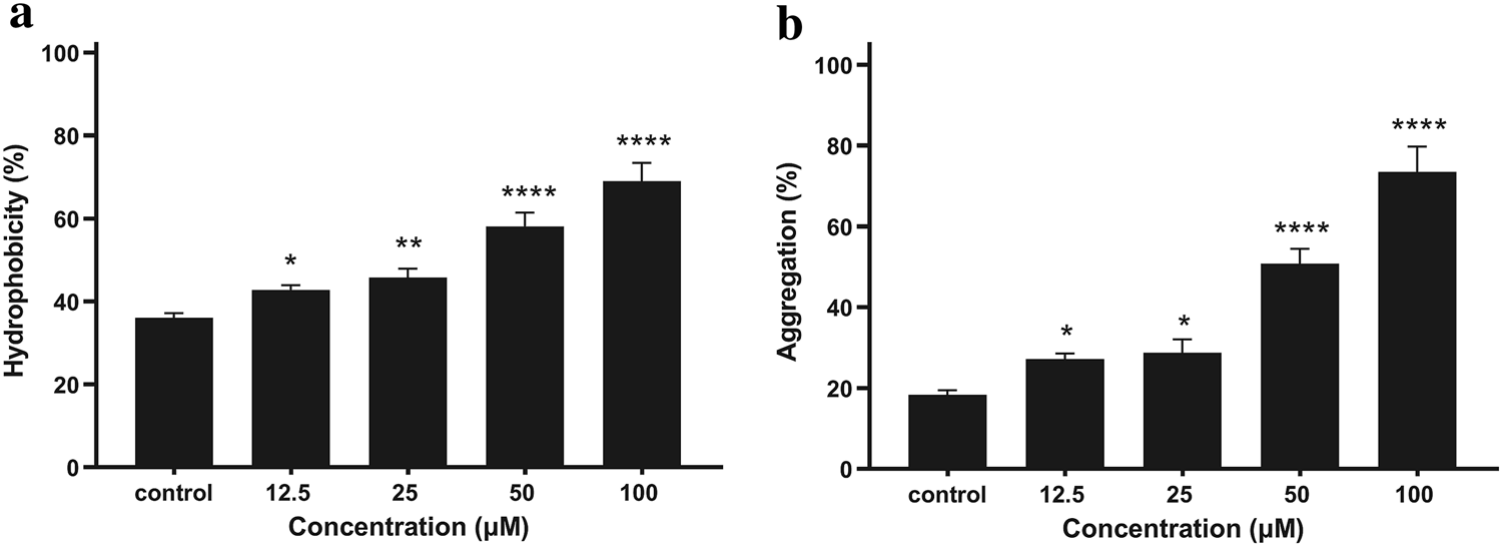
Effect of quercetin on hydrophobicity and aggregation. (**a**) Bacterial cells cultured in BHI with quercetin were washed and re-suspended in PUM buffer. 0.4 mL of hexadecane was added to 2 mL of the cell suspensions in tubes. After vortexed for 60 s and then incubated for 15 min at room temperature, the OD_550nm_ of the aqueous phase was measured and the hydrophobic activity was calculated. (**b**) *P. gingivalis* were cultured anaerobically at 37 °C for 48 h in BHI broth, harvested and re-suspended in PBS to an OD_600nm_ of 1.0. After 3 h, aggregation was calculated by measuring the decrease in OD_600nm_ of each suspension at 37 °C. Bars denoted by (*), (**) and (****) indicate significant difference at *p* < 0.05, *p* < 0.01 and *p* < 0.0001, respectively by one-way analysis of variance (ANOVA) with Dunnett’s post hoc test.

The hydrophobicity of the bacterial surfaces was determined by measuring the percentage of their adherence to hydrocarbons. The hydrophobicity assay revealed that quercetin increased *P. gingivalis* surface hydrophobicity in a dose-dependent manner as shown in Fig. 6a. The surface hydrophobicity rates with different concentrations of quercetin (12.5, 25, 50, and 100 μM) were 42.78 ± 1.09% (P = 0.044), 45.79 ± 2.09%, 58.10 ± 3.30%, and 68.95 ± 4.41%, respectively; which were substantially higher than that of the control group (36.06 ± 1.16%).

We compared the aggregation of *P. gingivalis* with different concentrations of quercetin. As shown in Fig. 6b, the aggregation rate reached 18.35 ± 1.13% after 3 h incubation. There was a dose-dependent increase in bacterial aggregation with different concentrations of quercetin. With increasing concentrations of quercetin, the aggregation rate increased from 27.18 ± 1.38% at 12.5 μM to 73.49 ± 6.25% at 100 μM.

### Effect of quercetin on gene expression

**Figure 7:**
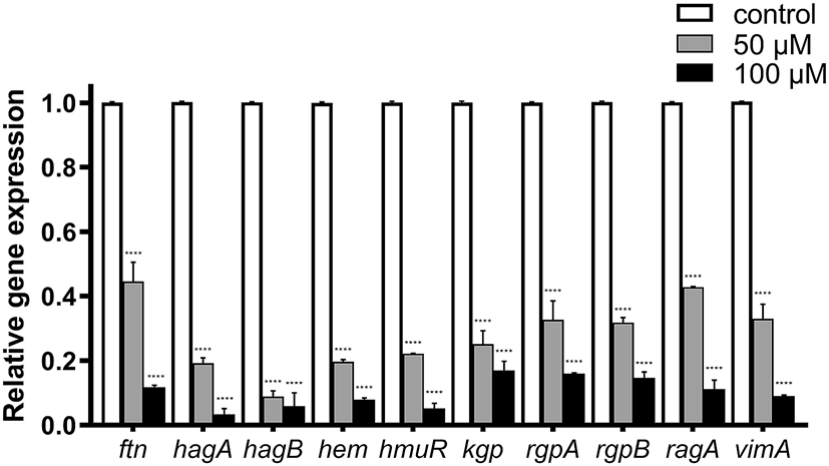
Effect of quercetin on gene expression. Transcriptional levels of the virulence genes in *P. gingivalis* cultured in BHI supplemented with 50 and 100 μM quercetin were detected by real time-PCR. Bars denoted by (****) indicate significant difference at *p* < 0.0001 by one-way analysis of variance (ANOVA) with Dunnett's post hoc test.

To gain insight into virulence factors and iron/heme utilization-related gene expression, real-time PCR analysis was used to quantify the effect of quercetin on *P. gingivalis*. As shown in Fig. 7, a significant and dose-dependent decrease expressions of all tested gene were observed. Among them, the virulence factor genes were including *hagA*, *hagB*, *hem* (involved in hemagglutination); *kgp*, *rgpA*, *rgpB* (involved in gingipain); *ragA* (immunodominant surface proteins), and *vimA* (virulence modulating gene A). Expression levels in virulence genes with quercetin were reduced ranging from 0.428- to 0.0884-fold at 50 μM and from 0.170-fold to 0.0340-fold at 100 μM. After investigating the effect of the quercetin on the expression of virulence factor genes, we also evaluated their effect on iron acquisition and metabolism (*hmuR* and *ftn*). At a concentration of 50 μM quercetin treatment, the expression of *hmuR* and *ftn* decreased 0.222-fold and 0.446-fold, respectively. More specifically, quercetin significantly inhibited the expression of both *hmuR* (0.0518-fold) and *ftn* (0.118-fold) at a concentration of 100 μM. The results indicate that quercetin inhibited the expression of virulence factors and iron/heme utilization-related genes in *P. gingivalis*.

## Discussion

*P. gingivalis* which detected with greater frequency and at higher levels at periodontal sites is strongly associated with periodontal disease and has been considered as a major etiologic agent for periodontitis^22,23^. In the present study, we determined the effect of quercetin on gingipain activities, hemagglutination activity, hemolytic activity, biofilm formation, hydrophobicity and aggregation, which all contribute to *P. gingivalis* virulence factors. We also studied its effect on the expression of *P. gingivalis* various virulence factor genes by RT-PCR. Firstly, we detected the potential antibacterial activity of quercetin against planktonic *P. gingivalis*. The MIC and MBC studies showed the lowest concentrations to inhibit or hamper planktonic bacteria growth was 200 and 400 μM respectively, which indicate that quercetin have both bacteriostatic and bactericidal effects. Growth curve assay confirmed that quercetin did not affect the growth of *P. gingivalis* under the concentration of 200 μM (Fig. S1). Previous study found that flavonoids can generate hydrogen peroxide that damages membranes to inhibit or hamper bacterial activity^24^. Therefore, we investigate the morphological changes in cell structure by TEM and our results confirmed that quercetin damaged the cell membrane and lead to cell death. To the best of our knowledge, it is the first time to systematically investigate the effect of quercetin at sub-MIC concentration on *P. gingivalis* virulence and pathogenic properties.

Gingipains degrade a large variety of host proteins such as immunoglobulins, extracellular matrix proteins and bactericidal proteins^25^. Some of them are providing nutrients and growth factors like iron, peptides and amino acids; while others result in disrupt the host immune defense mechanisms by modulating cytokine functions, degrading immunoglobulin, complement proteins and cell membrane proteins^26,27^. Thus, based on the above factors, inhibition of bacterial gingipains by quercetin may potentiate its therapeutic effects in the therapy of periodontitis. Our data showed that without affecting bacteria growth, sub-MIC concentrations of quercetin could significantly inhibit gingipains activities of *P. gingivalis* cell (Fig. 2) and gingipain extracts (Fig. S2) which was consistent with previous study^28^. And molecular docking analysis were carried out to confirm that quercetin could accommodate the binding pocket of both Kgp and RgpB by well shape matching and favorable hydrogen bond and van der Waals interactions, suggesting that quercetin is capable to inhibit the bioactivity of Kgp or RgpB by occupying the catalytic binding site. These results suggested that quercetin may be beneficial to prevent or slow down periodontal tissue destruction by inhibiting the gingipain activity of *P. gingivalis*.

*P. gingivalis* is an obligate anaerobe that efficiently utilizes heme, transferrin, and hemoglobin as sources of iron (essential nutrition) for bacterial growth and can acquire heme on its cell surface, resulting in black-pigmented colonies on blood agar plates^29^. Unlike other microorganisms, *P. gingivalis* does not possess a siderophore scavenging system for iron uptake or the enzymes required for heme biosynthesis^30^. It employs alternative mechanisms to acquire heme directly from erythrocytes. The mechanism of hemin acquisition from erythrocytes is a defense mechanism of *P. gingivalis* against reactive oxygen species in the oral inflammatory microenvironment and a mechanism for iron storage^31^. The mechanism involves hemagglutination, hemolysis, binding, and degradation of the hemoglobin molecule^32,33^. To determine the effect of quercetin in heme acquisition, we choose hemagglutination and hemolysis assay as targets to investigate the potential inhibitory activities. Our results showed that quercetin inhibited hemagglutination and hemolytic activity. These activities were also related to hemagglutinin-adhesin domains of gingipains, which participate the maturation of hemoglobin-binding receptor protein and may modify erythrocyte surface molecules^34^. Gingipain deficient mutants resulted in decreased hemagglutination and hemolytic activities compared with the wild-type strain^25,35^. Hemagglutinin may not only mediate the adsorption and invasion of bacteria into host cells but also agglutinate and lyse erythrocytes to survive in vivo^36^. The development of compounds against *P. gingivalis* hemagglutinins could be a promising cytoprotective strategy to prevent the harmful effects of long-term bacterial infection^30^. Therefore, quercetin may be significant in controlling the pathogenic potential of *P. gingivalis* through interference with mechanisms involved in hemagglutination and hemolysis.

In most environments, bacteria exist mainly in the form of biofilms^37^. Developing biofilm communities is an adaptive strategy for the survival of bacteria in host environments^38^. The biofilm phenotype is physiologically and functionally distinct from the planktonic bacteria. Bacteria enclosed in the biofilm structure can tolerate antibiotics and antibacterial agents more than 100–1000 times higher than planktonic bacteria and protect bacteria from attack by the host immune system, which enables persistent infection^38–40^. *P. gingivalis* has the ability to form biofilm, matrix-enclosed structure in subgingival plaque. Thus, we studied the effects of quercetin on *P. gingivalis* biofilms by crystal violet and MTT assays. Crystal violet assay was used for quantification of biofilm biomass, while the MTT assay was utilized to evaluate the metabolic activities of viable bacteria in biofilms. We found that biofilm formation was significantly decreased by quercetin at sub-MIC concentrations in this study. Confocal imaging also confirmed that the biofilms were clearly thinner and sparser with increasing concentrations of quercetin. These results showed that quercetin show inhibitory effects on *P. gingivalis* biofilm formation.

Previous studies demonstrated that quercetin can alter the cell surface properties to inhibit biofilm formation^41–43^. Thus, we assessed the cell surface properties by cell surface hydrophobicity and aggregation assays. Cell surface hydrophobicity is an important attribute of bacteria that contributes to adhesion and biofilm formation, and is influenced by the growth medium, bacterial age, and bacterial surface structures^44^. Our results showed that quercetin increased cell surface hydrophobicity and may lead to the inhibition of biofilm, which was consistent with previous study^45^. During biofilm development, autoaggregation is a process through which a strain within the biofilm produces polymers to boost the integration of genetically identical strains^46^, and is also important for the bacterium to attach in the oral cavity, which are essential for the pathological process leading to periodontitis. Our results showed that quercetin increased aggregation but decreased biofilm formation. CLSM fluorescence imaging also confirmed that *P. gingivalis* cells tended to aggregate into distinct clusters easily discernable after adding quercetin. These results were in agreement with our previous study concerning the effect of resveratrol on *F. nucleatum* aggregation and biofilm formation^46^. Besides, *P. gingivalis* aggregation is associated with cell wall components and characteristics such as hydrophobicity^47^, which may be the reason of quercetin to influence bacterial aggregation.

In addition, real-time PCR analysis was performed to evaluate the effect of quercetin on the gene expressions involved in virulence of *P. gingivalis*. Our results showed that the expressions of all selected virulence genes were down-regulated in the presence of quercetin. Among them, *rgpA*, *rgpB*, *kgp* genes are involved in gingipain and *hagA*, *hagB*, *hem* genes are involved in hemagglutination, which are major virulences of *P. gingivalis*. *rgpA* gene is related with bacterial surface proteins, for example, fimbrillin and Kgp^48^. *rgpB* gene is responsible for the production of gingipains and important in the pathogenesis of periodontal disease^49^. The *hagA*, *hagB*, *hem* genes facilitate the acquisition of heme from erythrocytes and other host cells which is necessarily for bacterial growth^50,51^. HagA and HagB also involved in adhesion to human coronary artery endothelial cells and gingival epithelial cells^51,52^. *RagA* is immunodominant surface antigens found in the serum of patients with periodontal disease; and can be stimulated when *P. gingivalis* is exposed to cotinine, nicotine, and cigarette smoke extract^53,54^. The *vimA* (virulence modulating) gene plays a role in acetyl coenzyme A (acetyl-CoA) transfer and modulates lipid A and its associated proteins. It also has multifunctional properties including oxidative stress resistance, glycosylation and anchorage of several surface proteins, protein sorting and transport^55,56^. Previous studies found that a *vimA* deficient strain increased aggregation and changed membrane surface proteins^57^. The down-regulation of *vimA* gene expression caused by quercetin may result in increasing *P. gingivalis* aggregation, which was consistent with our results. But there was no change in the hydrophobicity or ability to form biofilm in *vimA* deficient strain which was inconsistent with our results. These different results might be due to different methods used to test hydrophobicity and biofilm formation. Besides, another explanation for this observation may be that the effects of quercetin on hydrophobicity and biofilm formation have no relationship with *vimA* gene. In our study, the genes involved in iron acquisition and metabolism (*ftn* and *hmuR*) were also down-regulated by quercetin. Ferritin, encoded by the *ftn* gene, is one of the intracellular iron-storage proteins and particularly important for *P. gingivalis* to survive under iron deprived conditions^58^. HmuR (heme/hemoglobin utilization receptor HmuR), which has homology with TonB-dependent outer membrane heme/hemoglobin receptors is utilized for hemin and hemoglobin binding and hemin transport^59,60^. Therefore, quercetin inhibited the expression of virulence factors and iron/heme utilization-related genes at the transcriptional level.

Previous studies have investigated antimicrobial-agents-containing mouth rinse for *P. gingivalis* in clinical studies^61,62^. The essential-oils mouth rinse group containing thymol could significant reduce the occurrence of *P. gingivalis* in saliva, and present reductions of pocket depth (PPD), plaque index (PI), and gingival index (GI) comparing baseline at 45 (T1) and 180 (T2) days after periodontal therapy^61^. Another mouth rinse containing *Enteromorpha linza* extract (consist of polyphenol) significantly inhibits *P. gingivalis*, and reduces plaque, improves the condition of gingival tissues, and reduces bleeding^62^. This study provides theoretical basis for application of quercetin, a natural polyphenol, in the clinical perspective.

In conclusion, our study demonstrated that quercetin inhibits virulence and physiological properties of *P. gingivalis* including gingipain activity, hemolytic activity, hemagglutination activity, biofilm formation, hydrophobicity, aggregation, and virulence gene expression. Our study provides new evidence that quercetin impair the pathogenicity of *P. gingivalis* and might be useful in treatment of periodontal disease. Further studies are required to better understand the molecular mechanism underlying of quercetin inhibition on *P. gingivalis* pathogenicity.

## Materials and methods

### Bacteria and culture conditions

*Porphyromonas gingivalis* ATCC 33277 was provided by Laboratory of Oral Microbiota and Systemic Diseases, Shanghai Ninth People’s Hospital, Shanghai Jiao Tong University School of Medicine and grown in Brain Heart Infusion Broth (BHI; Difco Laboratories, Sparks, MD, United States) supplemented with hemin (5 μg/mL), menadione (0.5 μg/mL) at 37 °C under anaerobic conditions (80% N_2_, 10% CO_2_, and 10% H_2_).

### Bacteria antibiotic susceptibility assay

The drug susceptibility of planktonic cultures was determined by a broth microdilution assay. The quercetin (Sigma-Aldrich, St.Louis, MO, United States) was dissolved in dimethyl sulfoxide (DMSO, Sigma-Aldrich, St.Louis, MO, United States) and diluted at final concentrations of 0–1600 μM. A 20 μL quantity of *P. gingivalis* suspension (1 × 10^7^ CFU/mL) was added in a flat-bottomed 96-well microplate and incubated under anaerobic conditions at 37 °C. A blank control and a vehicle control were also prepared. The minimal inhibitory concentration (MIC) referred to the lowest concentration of quercetin that inhibited visible bacterial growth. To determine the minimal bactericidal concentration (MBC) values, an aliquot of 10 μL cell suspension from each well was taken, and bacterial clones were counted after incubation for 3–5 days. The MBC was defined as to the lowest concentration at which no bacterial growth observed on the blood agar plates.

### Transmission electron microscopy

Morphological changes in the cells were observed by transmission electron microscopy (TEM). The *P. gingivalis* cells with different concentrations of quercetin cultured at 37 °C for 4 h. The cell pellets were washed with PBS and fixed with 2.5% glutaraldehyde at 4 °C. Then, they were exposed to 2% osmium tetroxide for 2 h, dehydrated in a series of ethanol (30%, 50%, 70%, 85%, 95%, 100% and 100%) and dried in acetone solutions three times (50%, 100% and 100%) for 15 min each. Then the samples were embedded in resin blocks, cut into ultrathin (70-nm) sections, and stained with uranyl acetate and lead citrate. Each specimen was examined by TEM (FEI Talos L120C).

### Gingipain activity assays

Gingipain activity was measured according to the previous methods with minor modifications^63^. Briefly, a 24 h *P. gingivalis* culture was harvested by centrifugation, washed, and suspended in PBS to an optical density at 660 nm of 2 for Rgp activity and of 1 for Kgp activity. *P. gingivalis* cells were incubated in PBS with or without quercetin, a specific substrate for Rgp (0.4 mM, N-α-benzoyl-dl-arginine-*p*-nitroanilide, BAPNA) or Kgp (0.4 mM, acetyl-lysine-*p*-nitroanilide, ALNA). The mixtures were incubated at 37 °C in the dark for 3 h. Activity of Rgp and Kgp indicated as the hydrolysis of the Rgp and Kgp-specific chromogenic substrates was detected at every 2 min for 1 h by measuring the absorbance at 405 nm. The assay was repeated three times independently to ensure reproducibility.

### Molecular docking

Structures of Kgp (PDB: 6I9A) and RgpB (PDB: 1CVR) were retrieved from the Protein Data Bank. Proteins were prepared using the Protein Preparation Wizard in Maestro (Schrodinger, Inc., version 10.2). The crystal structures were minimized using the OPLS_2005 force field with the maximum root mean square deviation (RMSD) value of 0.3 Å. The ligands were prepared with the LigPrep module in Maestro, including adding hydrogen atoms, ionizing at a pH range from 5.0 to 9.0, and producing the corresponding low-energy 3D structure. The prepared compound was docked into the active site of Kgp/RgpB using Glide with default settings. The docked poses were ranked by Gscore, and the one with the lowest binding energy was selected for binding mode analysis.

### Hemagglutination assays

The hemagglutination assays were performed as previously reported^47^. Briefly, overnight cultures of *P. gingivalis* with quercetin were harvested, centrifuged, and suspended in PBS. Then, 100 μL of PBS was added to each well of a 96-well round-bottom microtiter plate, 100 μL of the bacterial cells was added to the first well and serially diluted (1:2 to 1:128). At the same time, sheep erythrocytes were washed and re-suspended in PBS. Finally, sheep erythrocytes suspension (100 μL, 2% in PBS) and quercetin were added to each well, and the plate was incubated at room temperature for 3 h. Hemagglutination activities were evaluated visually. Assays were repeated three times independent experiments.

### Hemolytic activity assays

Hemolytic activity was performed as previously reported with minor modification^64^. In brief, *P. gingivalis* cells were centrifuged, and re-suspended to a final OD_600nm_ of 1.5 in PBS. Fresh sheep erythrocytes (Beiruite Bio-technology, Zhengzhou, China) were harvested by centrifugation from whole blood and washed with PBS until the supernatant did not contain hemoglobin pigment visibly. The sheep erythrocytes at a concentration of 1% were mixed with an equal volume of bacterial cells with or without quercetin at 37 °C for 18 h. The hemolytic activity was determined at wavelength of 405 nm. The means and standard deviations of three independent experiments were calculated.

### Biofilm formation assays

Biofilm biomass was examined by the crystal violet staining method with minor modification^13^. *P. gingivalis* suspension was grown in BHI supplemented with quercetin for 48 h at 37 °C in a 96-well polystyrene plates. The culture supernatant was removed and washed three times with sterile PBS. Then, the adherent biofilms were incubated with methanol for 15 min followed by staining with 0.04% (w/v) crystal violet for 15 min. After washing with deionized water, 95% ethanol was added to detect. The OD values were recorded at wavelength of 550 nm.

The effect of quercetin on the viability of biofilm was determined using the 3-(4,5-dimethylthiazolyl-2)-2,5-diphenyltetrazoliumbromide (MTT) method^65^. The biofilm was formed as described for the above method. MTT (0.5 mg/mL) was added to detect the metabolic activity of the biofilms. The plates were cultured for 3 h in a dark place at 37 °C. Following incubation, MTT solution was gently aspirated from each well, and 100 μL of lysing solution was added to dissolve the formazan crystals. The absorbance of the solution was measured at a wavelength of 590 nm by microplate reader. All experiments were performed in triplicate with at least three replicates, and wells without cells were used as blank controls.

### Biofilm structure observation

*P. gingivalis* biofilms were formed as described above assay for 48 h on glass coverslips with different concentrations of quercetin. Bacterial cells were washed three times with saline to remove unbound cells and stained with the LIVE/DEAD BacLight Bacterial Viability Kit (Molecular Probes Inc., Eugene, Oregon, United States). After staining for 30 min in the dark, CLSM (Leica TCS SP8; Leica Microsystems, Wetzlar, Germany) was applied to acquire images and measure the thicknesses of biofilm. The exciting laser intensity, background level, contrast and electronic zoom were fixed for each experiment. In each experiment, at least five random fields were recorded.

### Hydrophobicity assays

The relative surface hydrophobicity was determined by measuring adherence to n-hexadecane^66^. Briefly, bacterial cells cultured with quercetin were washed in phosphate urea magnesium (PUM) buffer and re-suspended in the same buffer. Then, 0.4 mL of hexadecane (Sigma-Aldrich, St. Louis, MO, United States) was added to 2 mL of the cell suspensions in tubes. This mixture was vortexed for 60 s, and then incubated for 15 min at room temperature. The OD_550nm_ of the aqueous phase was measured, and the hydrophobic activity was calculated using the formula: [(OD_550nm_ before mixing) − (OD_550nm_ after mixing)]/(OD_550nm_ before mixing) × 100%. Independent experiments were repeated three times to verify the results.

### Aggregation assays

Aggregation assays of *P. gingivalis* was performed as described previously with slight modification^67^. Briefly, *P. gingivalis* were cultured anaerobically in BHI broth, and harvested by centrifugation and re-suspended in PBS to an OD_600nm_ of 1.0. After 3 h, aggregation was monitored by measuring the decrease in OD_600nm_ of each suspension at 37 °C. The percentage of aggregation was calculated by the following equation: Aggregation rate = (OD_Initial_ − OD_3 h_)/(OD_Initial_ − OD_Blank_) × 100%. Bacterial aggregation assays were performed in triplicate independent experiments.

### Quantitative analysis of gene expression by RT-PCR

The total RNA of *P. gingivalis* was extracted using Trizol reagent (Takara, Dalian, China), following the protocols provided by the manufacturer, and measured by Nanodrop 2000 to determine the RNA concentration. Reverse transcription was performed by cDNA synthesis kit (Takara, Dalian, China) to generate cDNA. Amounts of mRNA transcripts were measured by the Roche LightCycler 480 real-time PCR detection system (Roche, Basel, Switzerland). Reactions were performed with 20 μL of a mixture containing 10 μL of SYBR Premix (2 ×; Takara, Dalian, China), 1.6 μL of each primer, 3.4 μL of water and 5 μL of the cDNA template. The forward and reverse primer sequences are shown in Table 1^68,69^. Real-time PCR conditions included 30 s at 95 °C; 10 s at 95 °C, 20 s at 60 °C and 15 s at 72 °C for 40 cycles. The fold changes in gene expression were using the ΔΔCt method. Each assay was performed with three independent RNA samples. The results correspond to three experiments independently.

**Table 1:** Nucleotide sequences of primers used in this study^68,69^.

Gene*	Description	Primer sequence (5′-3′)	
		Forward	Reverse
*16S rRNA*	Normalizing internal standard	TGTAGATGACTGATGGTGAAA	ACTGTTAGCAACTACCGATGT
*ftn*	Ferritin	CGGCGAGGTGAAGATAGAAG	CTCCTGAGAGAGACGGATCG
*hagA*	Hemagglutinin protein HagA	TAAATAAGGGCGGAGCAAGA	GACGGAAAGCAACATACTTCG
*hagB*	Hemagglutinin protein HagB	TGTCGCACGGCAAATATCGCTAAAC	CTGGCTGTCCTCGTCGAAAGCATAC
*hem*	Hemolysin	ACGAAGCCTTGTTCTCCTCA	CAATGAATATGCCGGTTTCC
*hmuR*	Heme/hemoglobin utilization receptor HmuR	CTCCCATGCGGCCAACCCTCC	GCAGACGGGCTGTACGGCTACC
*kgp*	Lysine-specifc cysteine proteinase Kgp	AGGAACGACAAACGCCTCTA	GTCACCAACCAAAGCCAAGA
*rgpA*	Arginine-specifc cysteine proteinase RgpA	CACCGAAGTTCAAACCCCTA	GAGGGTGCAATCAGGACATT
* rgpB*	Arginine-specifc cysteine proteinase RgpB	GCTCGGTCAGGCTCTTTGTA	GGGTAAGCAGATTGGCGATT
*ragA*	Receptor antigen A	CGCTATTCTTCCTTTGCTTGCT	GATCGTGGTGTTTCCGACAA
*vimA*	Virulence modulating gene A	TCGCGTAGTCTGAGAGTAACCTT	GGTATAAACGAAGACAGCACGAC

### Statistical analysis

All data were presented as means ± SD. Statistical analysis was performed with one-way analysis of variance (ANOVA) with Dunnett’s post hoc test. All statistical analyses were carried out using SPSS software (SPSS 15.0 software, United States). The difference was considered statistically significant at P < 0.05.

## Supplementary information

Supplementary Information.

## Data Availability

All data generated or analyzed during this study are included in this published article.
