# Supplementary material for: Quercetin inhibits virulence properties of Porphyromas gingivalis in periodontal disease
Source: Sci Rep. 2020 Oct 27;10:18313. doi: 10.1038/s41598-020-74977-y (PMC7591570; doi:10.1038/s41598-020-74977-y)

Supporting Information

Figure S1. Growth curve of planktonic *P. gingivalis* with different concentrations of quercetin.

The overnight culture of *P. gingivalis* was used to anaerobically inoculate a fresh BHI culture with different concentrations of quercetin (0, 12.5, 25, 50, 100, and 200 μM) at 37°C anaerobically for 48 h. The optical density at 600 nm (OD_600nm_) was measured by a spectrophotometer (UV1601, Shimadzu, Japan) every 4 h throughout incubation. Each experiment was performed with triplicate samples at each time point. The results correspond to three experiments independently.


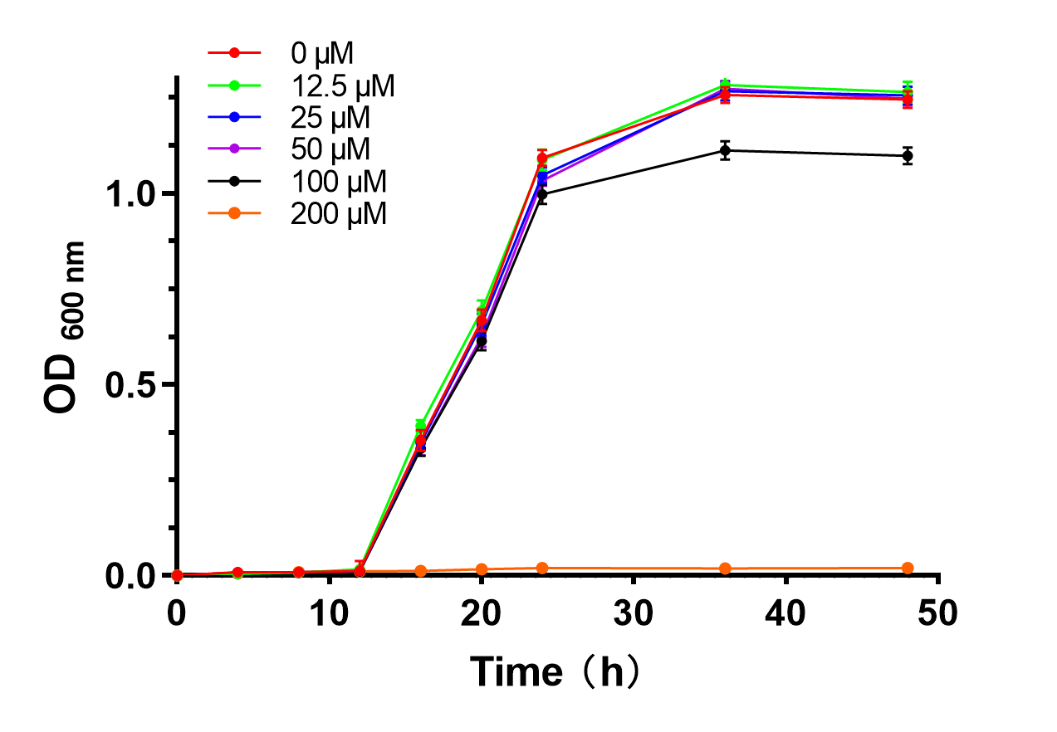


Figure S2. Effect of quercetin on Arg-gingipain (Rgp) and Lys-gingipain (Kgp) activity for *P. gingivalis* gingipain extracts. Gingipain active extracts were prepared according to previously published protocols^63^. Briefly, *P. gingivalis* strain ATCC 33277 was grown in BHI. After removing *P. gingivalis* cells from the cultures by centrifugation, the extracellular culture fluid was precipitated in a 60:40 ratio of acetone to cell-free medium, with constant stirring over a period of 15 min below 0 °C. The precipitate was centrifuged and the pellet was resuspended for dialyzed. After dialysis, the sample was ultracentrifugation to extract gingipain. The *P. gingivalis* gingipain extracts were diluted 1:25 for Rgp activity and 1:5 for Kgp activity, and incubated with or without quercetin at 37°C in the dark for 3 h. Activity of Rgp (a, b) and Kgp (c, d) indicated as the hydrolysis of the specific chromogenic substrates (BAPNA and ALNA) was detected at every 2 min for one hour by measuring the absorbance at 405 nm.


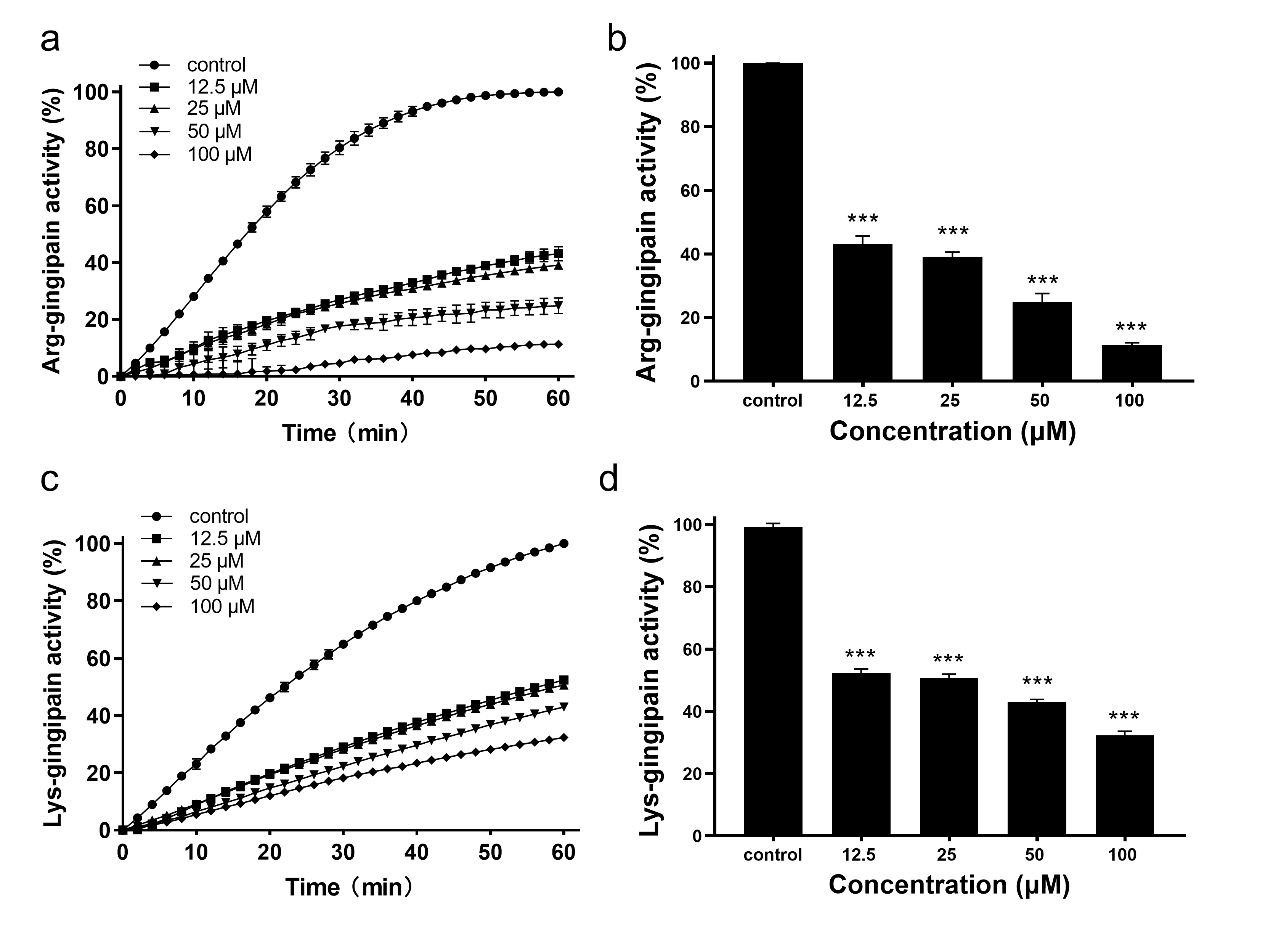

Supplement: Supplementary file 1 — Supplementary Information. [file 41598_2020_74977_MOESM1_ESM.docx]
